# Rhabdomyolysis After Prolonged Tourniquet Application Is Associated with Reversible Acute Kidney Injury (AKI) in Rats

**DOI:** 10.3390/biomedicines12112607

**Published:** 2024-11-14

**Authors:** Thomas J. Walters, Luciana N. Torres, Kathy L. Ryan, Robert V. Hainline, Stephanie M. Lipiec, Ijeoma E. Obi, Jennifer Ybarra, Casey E. Niland, Lusha Xiang

**Affiliations:** 1 US Army Institute of Surgical Research, JBSA Fort Sam Houston, San Antonio, TX 78236, USA; thomas.j.walters22.ctr@health.mil (T.J.W.); luciana.n.torres.ctr@health.mil (L.N.T.); kathy.l.ryan.ctr@health.mil (K.L.R.); stephanie.m.lipiec.mil@health.mil (S.M.L.);; 2 59th Medical Wing, JBSA Lackland Air Force Base, San Antonio, TX 78236, USA

**Keywords:** ischemic injury, crush injury, bone marrow, tourniquet shock, trauma, glomerular filtration rate

## Abstract

Extremity trauma, including ischemia (e.g., prolonged tourniquet application or crush), is common among battlefield injuries. Injured muscle releases toxins leading to rhabdomyolysis and, potentially, acute kidney injury (AKI). The goal of this study was to characterize sequelae of ischemic extremity injury over 72 h, focusing on time courses of rhabdomyolysis and AKI. Male Sprague Dawley rats were placed into two groups. Ischemic injury was produced in anesthetized rats using bilateral tourniquets (TK; n = 10) for 5 h; control (CON; n = 9) rats were treated identically without TK application. Indicators of rhabdomyolysis and renal function were measured in conscious rats 1 day preinjury (baseline, BL) and then at 1.5, 24, 48, and 72 h post-TK release. Prolonged TK application produced necrosis in both muscle and bone marrow but not in kidney. The wet/dry weights indicated edema in injured limbs at 72 h (4.1 (0.5) (TK) vs. 2.9 (0.1) (CON); *p* < 0.001). TK rats exhibited a 100-fold increase in creatine kinase activity compared to CON at 1.5 h (20,040 (7265) U/L vs. 195 (86) U/L (mean (SD); *p* < 0.0001). TK decreased the mean glomerular filtration rate (GFR; *p* < 0.001) at 1.5 h, but these values recovered by 24 h in concert with elevated urinary flow and alkalinization. Prolonged ischemic extremity injury therefore produced severe rhabdomyolysis without irreversible renal damage.

## 1. Introduction

Over the past 2 decades, tourniquets have reduced mortality in military and civilian settings. It has been estimated that between 1000 and 2000 lives were saved on the battlefields of Iraq and Afghanistan because of liberal tourniquet use [1] Potential complications caused by tourniquet-induced ischemia were largely avoided due to rapid evacuation to definitive medical care, with tourniquet removal generally taking place within 2 h of application [2]. While tourniquet application of 2 h or less is considered safe, increasing time progressively exacerbates muscle ischemia/reperfusion injury and systemic complications, in particular, rhabdomyolysis. Six hours (6 h) of tourniquet application is considered the maximum time a tourniquet can be applied to muscle while preventing possible life-threatening systemic complications, such as acute kidney injury (AKI). This fact has led to recommendations to leave tourniquets in place after 6 h of application effectively resulting in physiological amputation of the limb due to extensive muscle necrosis [3]. In the recent Ukraine conflict, liberal tourniquet use routinely exceeds 6 h, often in casualties without significant vascular injury, resulting in unnecessary ischemic injury and limb loss [4].

Following reperfusion of an ischemic limb(s), e.g., following tourniquet release, vascular repair, or extrication of an entrapped limb(s) (crush injury), the contents of the necrotic muscle enter the circulation, resulting in rhabdomyolysis. These muscle constituents include myoglobin, K^+^, other catabolites, and inflammatory mediators, as well as reactive oxygen and nitrosative species [5]. Together, these muscle byproducts may induce severe or life-threatening injuries, including AKI, cardiac arrhythmia, pulmonary injury, extremity compartment syndrome, and coagulopathic conditions [3]. It should be emphasized that rhabdomyolysis is not unique to ischemic injury caused by tourniquet application, as other forms of muscle trauma, especially (muscle) crush injury, also result in release of muscle contents and rhabdomyolysis. Indeed, the consequences and treatment of crush injuries are assuming more relevance for both military and civilian medicine as warfare and/or terrorist activity occurs in dense urban environments [6,7]. Importantly, the magnitude of systemic complications is a function of the mass of traumatized muscle; for example, prolonged use of a bilateral tourniquet creates more rhabdomyolysis than that of a unilateral tourniquet [8].

Rodent models to study the relationship between rhabdomyolysis and the development of AKI have been developed. For example, dehydration combined with intramuscular injection of tissue extract solutions such as glycerol has been widely applied to induce rhabdomyolysis [9]. However, such models do not accurately replicate the ischemia reperfusion process that occurs with physical injury such as prolonged tourniquet application or crush injury [10]. Furthermore, glycerol-based models invariably produce rhabdomyolysis and severe AKI; this model has traditionally been tailored to create AKI, and the severity of AKI can be altered by increasing the dose of glycerol [11]. However, AKI occurs in only a fraction of rhabdomyolysis patients, particularly in those more severely injured [12]. The operational relevance of such models to real-life trauma scenarios is thus questionable.

While a number of animal studies have been conducted to determine survivability as a function of tourniquet time using both uni- and bilateral tourniquets, to our knowledge, none have directly examined their impact on long-term renal function. The objective of this study was to characterize the impact of prolonged (5 h) bilateral tourniquet application on the development of rhabdomyolysis and its effect on renal function over the subsequent 3 days in conscious rats, with the aim of developing a platform that could be used to develop and test the effectiveness of no- or low-fluid treatments for rhabdomyolysis.

## 2. Materials and Methods

### 2.1. Animals

Male Sprague Dawley rats (Charles River, Wilmington, MA, USA) were shipped at 8–11 weeks of age (326–350 g) and acclimated in our vivarium for at least a 1-week period before surgery. During the acclimation period, rats were housed in pairs; rats were housed singly once moved into metabolic cages. Environmental conditions were controlled by a heating, ventilation, and air conditioning (HVAC) system that met the Institute for Laboratory Animal Research (ILAR) Guide recommendations. Conditions were monitored by a Watchdog Environmental Monitoring System, with room temperature and humidity maintained at 19–23 °C and 30–70%, and lights on from 6:00 to 18:00. Food (LabDiet 5001 Rodent Diet, St. Louis, MO, USA) and water were provided ad libitum. Research was conducted in compliance with the Animal Welfare Act, its implementing Animal Welfare Regulations, and the principles of the Guide for the Care and Use of Laboratory Animals. The Institutional Animal Care and Use Committee at the US Army Institute of Surgical Research approved all research conducted in this study (A-23-002). The facility where this research was conducted is fully accredited by AAALAC International.

### 2.2. Inclusion Criteria

To be included for data analysis, each rat must have undergone successful catheterization on Day 1 and survived the 5 h bilateral tourniquet (TK) application on Day 2. A rat was included if it survived TK application and the release thereof, even if it died or was moribund before the end of the observation period (Day 5). 

### 2.3. Overview of Experimental Timeline

**Figure 1:**
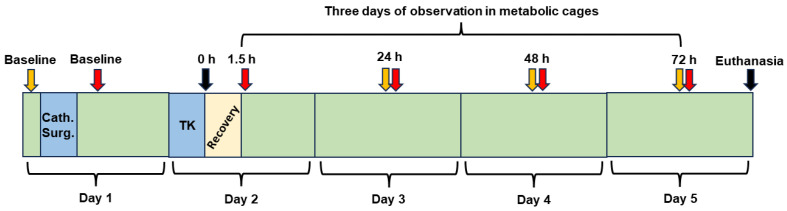
Timeline of the 5-day experimental protocol. The blue boxes represent the anesthetized periods (isoflurane), the yellow box represents the 1.5 h recovery from anesthesia after tourniquet (TK) treatment, and the green boxes represent conscious periods in the metabolic cages. The red arrows represent the time points for blood sample collections and measurements of mean arterial pressure (MAP), heart rate, and glomerular filtration rate (GFR), while the yellow arrows represent the time points for urine collection and measurements of water and food intake from the previous day. “Cath. Surg” represents the catheterization of the carotid artery on Day 1.

Figure 1 shows the timeline of experimental procedures. Rats were assigned to (1) control (CON; no TK) or (2) TK groups. Rats were placed into metabolic cages for acclimation 72 h prior to Day 1, the day of surgical implantation of a carotid catheter and a jugular vein catheter. Following catheterization, glomerular filtration rate (GFR), mean arterial pressure (MAP), and heart rate (HR) were measured, and both arterial blood and urine were sampled to establish baseline (BL) values. Rats were returned to the metabolic cages, and food and water consumption as well as urine output were measured throughout the remainder of the experiment. After 24 h (Day 2), rats were anesthetized, and a pneumatic TK was placed on each hindlimb for a period of 5 h. Anesthesia was discontinued, and GFR, MAP, and HR were measured in conscious rats 1.5 h after TK release. These parameters were also measured 24, 48, and 72 h later in conscious rats, and blood and urine were collected. Seventy-two hours after TK release (Day 5) (or earlier, if a rat was found to be moribund), rats were anesthetized and then euthanized with an intravascular injection of sodium pentobarbital (150 mg/kg body weight), and tissues were collected for pathological examination. CON rats were catheterized on Day 1 and anesthetized on Day 2 for the same time period required for TK placement and inflation, but TKs were not applied.

### 2.4. Day 1 Procedures—Catheterization and BL Measurements

Following 72 h acclimation in metabolic cages, rats were anesthetized with 1–3% isoflurane in 30% oxygen in preparation for catheterization of the carotid artery and jugular vein; anesthesia was maintained throughout the catheterization procedure. All animals received buprenorphine SR Lab (1.2 mg/kg, s.c.) as pre-emptive analgesia prior to catheter surgery. All surgical procedures were performed using an aseptic technique. An incision was made on the neck to expose and isolate a jugular vein and carotid artery. A catheter (PU-3Fr) was placed into the carotid artery, and a second catheter (PU-3Fr, Intramedic, BD, Franklin Lakes, NJ, USA) was placed into the jugular vein. Both catheters were exteriorized at the nape of the neck, and incisions were closed. A pharmaceutical-grade lock solution (heparin in 50% glycerol USP; Instech Laboratories, Inc., Plymouth Meeting, PA, USA) was used to keep the catheters patent throughout this study. The arterial catheter was used for daily blood pressure measurement and blood sample collection, while the venous catheter was used for solution administration (e.g., fluorescein-isothiocyanate (FITC)-labeled sinistrin). Anesthesia was discontinued, and rats were allowed to recover in a polycarbonate home cage.

At least 60 min later, rats were returned to the metabolic cage. A 1.0 mL arterial blood sample was taken, and the arterial catheter was attached to a blood pressure monitoring system equipped with a customized data acquisition system. After centrifugation and plasma collection, packed red blood cells were resuspended in sterile phosphate-buffered saline (PBS) and were reinfused via the jugular vein catheter to maintain oxygen-carrying capacity. Baseline urine was also collected from the metabolic cage. Arterial blood pressure measurements were made for 45 min. Noninvasive GFR assessment was begun as fully described below.

### 2.5. Tourniquet Injury (Day 2) 

Rats were first weighed to determine FITC-sinistrin dose. Rats were then anesthetized with 1–3% isoflurane in 30% oxygen and remained anesthetized throughout the TK application. The procedure for TK application has been previously described [13,14]. Briefly, the fur on both hindlimbs was clipped. Hindlimbs were elevated above the level of the heart for 5 min, and commercially available pneumatic digital cuffs (Model UDC 2.5, D.E. Hokanson, Inc., Bellevue, WA, USA) were placed as proximally as possible on both hindlimbs. TKs were inflated with a rapid cuff inflator equipped with a pressure transducer (Delfi Medical Innovations, Inc., PTSii Tourniquet Systems, Vancouver, BC, Canada) and maintained at a pressure of 400–420 mmHg. Immediately after TK inflation, hindlimbs were lowered, and TK pressure was maintained for 5 h. At 5 h, TKs were immediately deflated. CON rats were anesthetized for the same time period as the TK group but did not receive TK application. Rats were returned to the metabolic cage and allowed to recover for approximately 1.5 h. An infrared thermal source was positioned above each metabolic cage and connected to a thermometer and a thermostat to maintain ambient temperature between 27 and 28 °C from Day 1 until the last day of the experiment. 

### 2.6. Day 1–Day 4 Procedures

Following 1.5 h of recovery, blood and urine were sampled, and arterial blood pressure and GFR measurements were made (see Figure 1). The same procedures were performed at 24 and 48 h post-tourniquet release. 

### 2.7. Terminal Procedures (Day 5 or upon Early Moribund Status)

Rats were assessed at least twice daily following Day 1 procedures. If a rat was found to be moribund in consultation with the veterinary staff, terminal procedures were initiated before Day 5. For those rats surviving to Day 5, arterial blood and urine samples were obtained. Arterial blood pressure and GFR measurements were then made as at Day 1 in conscious rats. Rats were then anesthetized (isoflurane) and euthanized by intravenous sodium pentobarbital (FatalPlus) infusion.

After euthanasia, the hindlimbs and kidneys were harvested. Edema formation in one hindlimb and one kidney was determined from the wet-to-dry weight ratio; dry weight was obtained from placement in an oven (45–50 °C) for a minimum of 3 days. The remaining kidney and hindlimb were collected and fixed in formalin for histopathological assessment. 

### 2.8. Transcutaneous GFR Measurement

GFR was measured noninvasively in conscious rats using the FITC-labeled sinistrin clearance technique [15]. A miniature fluorescence density optical detector (MediBeacon, Creve Coeur, MO, USA) was attached to the skin to transcutaneously record excretion kinetics of FITC-sinistrin after an injection via the jugular vein (5 mg/100 g body weight; NIC-Kidney; Mannheim Pharma & Diagnostics, Mannheim, Germany). At the end of the observation period, the optical device for GFR recording was read and analyzed using MediBeacon Studio V2 software (MediBeacon, Creve Coeur, MO, USA). The transcutaneous GFR was calculated using the half-life derived from the excreted curve using the established three-compartment kinetic model [16]. 

### 2.9. Blood Measures and Urinalysis

Arterial blood samples were analyzed using an i-STAT Handheld Blood Analyzer (Abbott, Princeton, NJ, USA). CG4+ and CHEM8+ cartridges provided blood urea nitrogen (BUN), creatinine (Cr), lactate, hemoglobin concentration, pH, bicarbonate, potassium, and sodium measurements. CK concentrations were determined on plasma samples using a Siemens Dimension EXL 200 Integrated Chemistry System (Siemens Medical Solutions USA, Malvern, PA, USA). Hematocrit (Hct) was obtained by direct measurement of the packed cell volume using a microhematocrit tube and microhematocrit centrifuge (StatSpin^®^, HemoCue, Brea, CA, USA). Urinalysis was performed using Multistsix Pro^®^ 10 LS strips and a Clinitek Urine Analyzer (Siemens, Malvern, PA, USA). Urine measures included Cr, pH, protein, glucose, and blood. 

### 2.10. Calculations

Physiological parameters were calculated as follows:

- Cr clearance (CCr) = (UCr × urine flow) ÷ Cr, where UCr is urine Cr and Cr is blood Cr;
- Protein excreted = urine protein centration × urine volume/bodyweight.

### 2.11. Pathology

Cross-sections (∼8 µm) of the limb (distal to tourniquet) and kidney were stained for hematoxylin and eosin. A semi-quantitative analysis of the cross-sections was performed by a board-certified veterinary pathologist blinded to the treatment group. 

### 2.12. Statistical Methods

Data were analyzed using GraphPad Prism 10.1.0. For most data, a two-way ANOVA involving treatment (CON, TK) and time (5 levels for most analyses) with repeated measures for time or a repeated-measures mixed-effects model (REML; in the case of missing data) with Geisser–Greenhouse correction was conducted. Multiple means comparisons within treatments across time were conducted using a Tukey’s multiple comparison test. For data obtained after euthanasia (e.g., wet/dry weight), unpaired *t*-tests were used. Data are presented as arithmetic means (standard deviation, SD), and statistical significance was considered attained at *p* < 0.05. 

## 3. Results

Seven of the nine CON rats survived the entire study period. One CON rat was euthanized due to surgical complications prior to injury, and a second one was excluded due to reasons unrelated to the treatment, leaving n = 7. Two of the original ten TK rats died. One was found dead in its cage during the evening health check on Day 1; a fourth rat was found pre-moribund during the same health check and was euthanized. Data collected from these two rats prior to death were used in the analyses. Some data collection was precluded due to facility closure during inclement weather.

### 3.1. Muscle Injury

**Figure 2:**
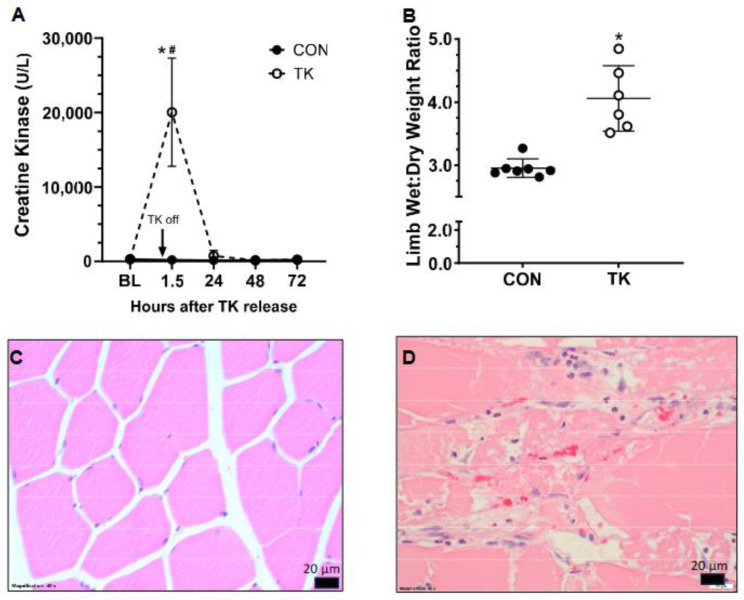
Parameters defining injury to hindlimb muscle following 5 h of tourniquet (TK) application. Panel (**A**), plasma creatine kinase (n = 4/group); Panel (**B**), limb wet/dry ratio (n= 7 and 6 for CON and TK, respectively); Panels (**C**,**D**), muscle from two rats (H&E, 40X). Panel (**C**) represents normal control muscle tissue. Panel (**D**) represents muscle from a rat following TK placement. Myocytes exhibit polyphasic degenerative and necrotic changes characterized by pale, swollen, and vacuolated sarcoplasm with disrupted myofibrils (degeneration); hypereosinophilic, shrunken, and fragmented or hyalinized sarcoplasm with loss of cross striations and a pyknotic or karyorrhectic nucleus (necrosis); or lightly basophilic sarcoplasm with multiple internalized, linearly arranged, vesiculate nuclei with prominent nucleoli (regeneration). Occasionally, multifocal random foci of inflammatory cells expand the epimysium, perimysium, and endomysium and surround and separate individual myocytes, composed primarily of neutrophils and histiocytes with rare lymphocytes and plasma cells. CON, control group; TK, tourniquet group; BL, baseline. * *p* < 0.05 between CON and TK groups at the time point; # *p* < 0.05 compared to BL within treatment group.

Five hours of bilateral TK application caused substantial muscle damage resulting in rhabdomyolysis, as indicated by a 100-fold increase in CK activity compared with CON on Day 1 (20,040 (7265) U/L vs 195 (86) U/L (*p* < 0.0001)). CK values then declined to near baseline by Day 2 (Figure 2A). TK resulted in a 37% greater muscle wet/dry weight ratio compared to CON (*p* = 0.0002), indicating substantial injury-related edema. Muscle injury and edema were further confirmed by histological analysis, which showed extensive myocyte degeneration and necrosis, as well as general edema (Figure 2C,D). Furthermore, TK injury produced necrosis of the bone marrow in four of the six hindlimbs examined (67%). There was no muscle or bone marrow damage noted in any hindlimb from CON rats (Supplemental Figure S1). 

### 3.2. Cardiovascular Function

**Figure 3:**
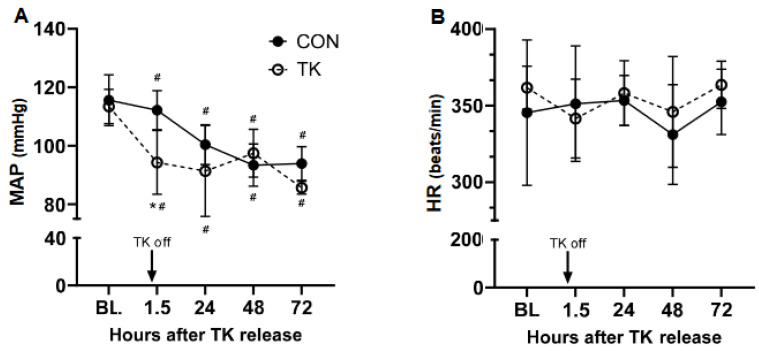
Systemic hemodynamics following 5 h of tourniquet application. Panel (**A**): MAP, mean arterial pressure; Panel (**B**): HR, heart rate. BL, baseline; CON, control group; TK, tourniquet group. The respective N for each group for both panels at BL, 1.5 h, 24 h, 46 h, and 72 h was 7, 7, 5, 7, 6 for CON and 8, 7, 6, 5, 5 for TK. * *p* < 0.05 between CON and TK groups; # *p* < 0.05 compared to BL within treatment group.

MAP declined (*p* < 0.0001) 1.5 h following TK release and remained significantly below BL for the remainder of the experiment (Figure 3A). MAP also decreased (*p* < 0.001) in CON by 24 h and remained significantly below BL through 72 h. MAP was lower in the TK than the CON group only at the initial post-treatment timepoint. Heart rate was not significantly affected by the treatment in either group (Figure 3B).

### 3.3. Renal Function

**Figure 4:**
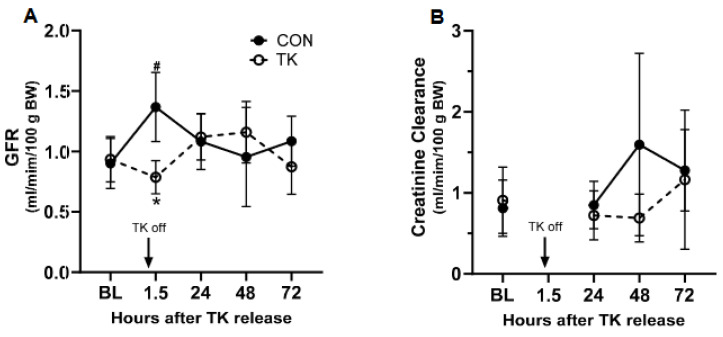
Parameters defining renal function and injury following 5 h of tourniquet (TK) application. Panel (**A**): Glomerular filtration rate (GFR) measured noninvasively using the FITC-sinistrin technique. The respective N for each group at BL, 1.5 h, 24 h, 46 h, and 72 h was 7, 7, 5, 7, 7 for CON and 8, 7, 6, 6, 6 for TK. Panel (**B**): Creatinine clearance normalized to body weight. The respective N for each group at BL, 24 h, 46 h, and 72 h was 7, 4, 6, 6 for CON and 8, 5, 5, 6 for TK. GFR, glomerular filtration rate; BL, baseline; CON, control group. * *p* < 0.05 between CON and TK groups; # *p* < 0.05 compared to BL within treatment group.

GFR differed between the TK and CON groups at 1.5 h post-treatment (*p* < 0.0001) due to a significant increase in GFR in the CON group (Figure 4A). CCr was unaffected by the treatment (Figure 4B). The kidney wet/dry weight ratios did not differ (*p* = 0.232) between the CON and TK groups. A pathological examination revealed tubular necrosis graded as mild in only one of the seven TK rats examined (Supplemental Figure S2). There were no other pathological changes noted in the examined kidneys.

BUN increased by 91% above BL 1.5 h following TK release (*p* < 0.0001) and remained 52% above BL at 24 h (*p* = 0.001), returning to BL by 48 h (Table 1). Plasma Cr followed a similar pattern with a 185% increase above BL at 1.5 h (*p* < 0.0001) in the TK group, a 72% increase above BL at 24 h (*p* = 0.006), and a return to baseline at 48 h (Table 1). BUN and Cr did not change over time in the CON group, resulting in significant differences between the groups at 1.5 and 24 h. No significant changes in BUN/Cre occurred due to the similar pattern of change between BUN and Cre during the observation period (Table 1). Urinary creatinine (UCr) was not significantly affected by the treatment in either group, and there was no difference in this parameter between the groups at any time point (unpublished findings).

### 3.4. Arterial Blood Measures

**Figure 5:**
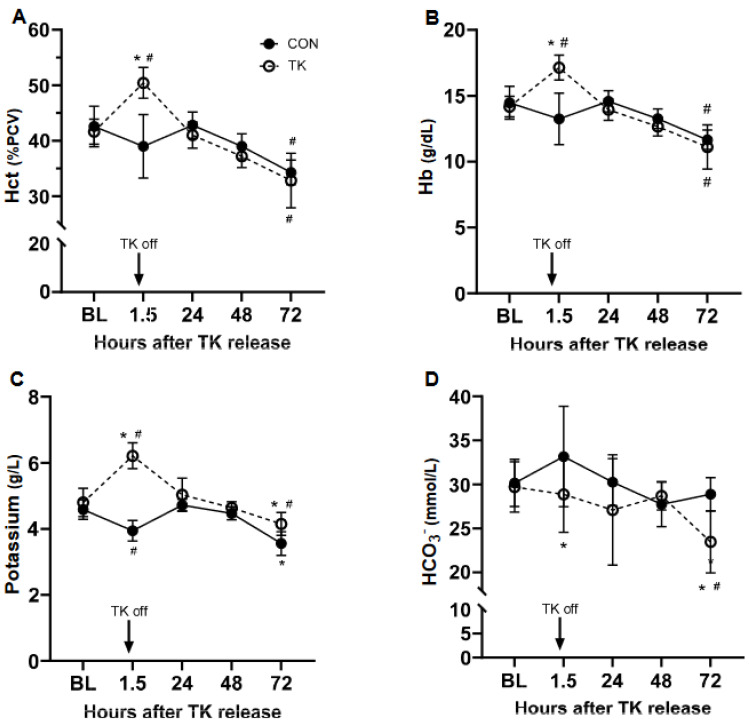
Blood parameters. Panel (**A**): hematocrit (HCT); Panel (**B**): hemoglobin; Panel (**C**): potassium; Panel (**D**): bicarbonate (HCO_3_^−^). BL, baseline; CON, control group; TK, tourniquet group; PCV, packed cell volume. * *p* < 0.05 between CON and TK groups; # *p* < 0.05 compared to BL within treatment group. The respective N for each group at BL, 1.5 h, 24 h, 46 h, and 72 h was 7, 7, 5, 7, 7 for CON and 8, 7, 6, 5, 6 for TK.

Following TK release, there was a 21% increase in Hct (*p* < 0.0001) followed by a decline to levels significantly below BL by 72 h (*p* < 0.0001) (Figure 5A). The Hct in the TK group was higher (*p* < 0.0001) than that in the CON group at 1.5 h after TK release. Hct values in CON were also significantly below BL at 72 h (*p* = 0.0002), and there were no differences between treatments at this time point. Changes in hemoglobin were identical in pattern and magnitude of change in both the CON and TK groups (Figure 5B). 

Blood potassium levels increased 29% above BL at the 1.5 h time point (*p* ≤ 0.0001) and then declined to a level significantly below BL at 72 h (*p* = 0.02) (Figure 5C). In CON, potassium decreased 13% at 1.5 h, returned to BL at 24 and 48 h, and then decreased by 20% at 72 h (*p* < 0.0001). The potassium level in the TK group was therefore greater than that in the CON group at the 1.5 and 72 h time points. The BL sodium level in the TK group was slightly lower than that in the TK group (Table 1). Sodium in the TK group decreased slightly at 1.5 h (*p* = 0.04), returning to BL by 48 h. There were no changes in sodium across time in the CON group. Sodium levels were lower in TK than in CON at each time point except at 72 h post-treatment. Calcium was mostly unaffected by injury except for a decline compared to CON at 72 h (*p* = 0.013). Bicarbonate levels were significantly different between TK and CON at 1.5 h (*p* = 0.035), primarily due to an elevation in CON (Figure 5D), and declined by 21% compared to BL at 72 h (*p* = 0.024), which was also significantly less than CON (*p* = 0.012).

Lactate levels doubled following TK (*p* = 0.004) and remained significantly above BL throughout the study period (Table 1). Lactate did not change across time in the CON group, and TK lactate was higher than that in the CON group at every time point. Blood pH was not significantly affected over time in either the TK or the CON group compared to BL. However, pH in the TK group was lower than that of the CON group at 1.5 (*p* = 0.027) and 48 h (*p* = 0.019) post-treatment. 

### 3.5. Water Consumption and Urine Measures

**Figure 6:**
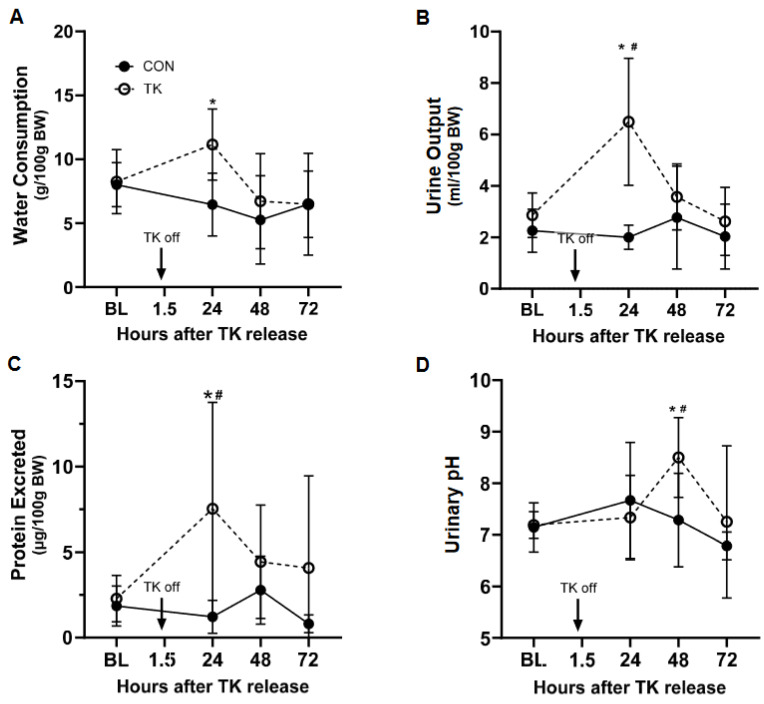
Water consumption (Panel (**A**)) and urinary parameters (Panels (**B**–**D**)). Panel (**B**): daily urine output, normalized to body weight (BW); Panel (**C**): urinary protein excretion, normalized to BW; Panel (**D**): urinary pH. BL, baseline; CON, control group (solid circle); TK, tourniquet group (hollow circle). * *p* < 0.05 between CON and TK groups; # *p* < 0.05 compared to BL within treatment group. The respective N for each group at BL, 24 h, 46 h, and 72 h was 7, 7, 6, and 7 for CON and 8, 6, 6, and 6 for TK, respectively. * *p* < 0.05 between CON and TK groups; # *p* < 0.05 compared to BL within treatment group.

Rats from both groups displayed a statistically similar pattern of body weight loss after treatment, which was below BL from 48 h (CON, *p* = 0.03; TK, *p* = 0.03) through to 72 h (CON, *p* = 0.04; TK, *p* = 0.04) (Supplemental Figure S3). There were no differences in body weight between the groups at any time point. The decline in body weight was likely due to a reduction in food consumption, which was significantly reduced relative to baseline in the TK group by 48 h (*p* < 0.0001) through to 72 h (*p* < 0.0001) (Supplemental Figure S3). Similarly, food consumption was significantly reduced at both 48 h (*p* = 0.04) and 72 h (*p* = 0.01) in the CON group. Since this occurred in both groups, the declines in food consumption and body weight were likely the result of the extended period of anesthesia (approximately 300 min). Daily water consumption did not significantly change from BL in either group during the period of observation but was greater in the TK group than in the CON group at 24 h post-TK release (Figure 6A). Urine output was elevated in the TK group 24 h after TK release (*p* < 0.0001) and was greater than CON (*p* < 0.0001) at that time point (Figure 6B). This diuresis after TK treatment was transient, however, with daily urine volume returning to BL levels by 48 h. Likewise, the amount of protein excreted in the urine was elevated over BL in the TK group 24 h after TK release (*p* = 0.040), while this did not occur in the CON group (Figure 6C). Protein excretion in the TK group was thus greater than that in the CON group at 24 h (*p* = 0.002), but this difference did not persist at later time points. Urine pH increased over BL levels in the TK group only 48 h after TK release (*p* = 0.014); urine pH was not changed from BL levels in the CON group, resulting in urine pH in the TK group being elevated compared to that in the CON group at the 48 h time point (*p* = 0.014) (Figure 6D). 

## 4. Discussion

The major findings of this study are the following: (1) bilateral TK application at 400 mmHg for 5 h caused severe rhabdomyolysis with ischemia-induced bone marrow damage in rats; however, (2) the severe rhabdomyolysis did not produce acute intrarenal injury as evidenced by only a transient decrease in GFR and no pathological changes to the kidney on histological examination. 

The amount of material released from injured muscle into the circulation is dependent on the duration of TK application and the mass of muscle affected. CK increases through 3 h of unilateral TK application with no further elevation through 8 h of ischemia and never exceeds 10,000 U/L [17], the clinical criterion for rhabdomyolysis. This suggests that, in rats, unilateral TK application does not involve adequate muscle mass to produce rhabdomyolysis. In pilot studies, we found that 4 h of bilateral TK application failed to raise CK above 10,000 U/L. However, 6 h of bilateral TK application resulted in 100% mortality, with most of the deaths occurring several hours after TK release (unpublished data). Therefore, in order to observe the effects of rhabdomyolysis on renal function over the subsequent 3 days in conscious rats, we selected 5 h of bilateral TK application for use in the current study. The rapid and massive increase in CK suggests that we successfully developed a rat model of TK-induced rhabdomyolysis, as no other condition except rhabdomyolysis can cause such extreme CK elevation [18,19]. The simultaneously increased potassium levels in the TK group also support massive tissue damage. 

In human patients, CK can rapidly increase immediately after muscle injury but declines at a slower and constant rate of 40–50% of the previous day’s level over subsequent days [20,21]. In contrast, we observed a rapid increase in CK (1.5 h post-TK release), followed by a rapid decline by 24 h post-release. One possible explanation for this difference is that TK application in humans is accompanied by direct traumatic damage to the distal muscle, and the injured muscle continues to release CK and other muscle constituents into the circulation over time [18]. In our study, there was no concomitant muscle or vascular injury necessitating the application of the TK, thus the release of CK may have been complete soon after restoration of blood flow. We also cannot discount the possibility that rats clear CK from blood faster than humans, although this has not been published to our knowledge. Nevertheless, rat models offer the ability for invasive measurements and access to GFR measurement, which is important given that AKI is a major complication of rhabdomyolysis. 

In addition to the high levels of CK, the limb edema, muscle degeneration, and necrosis identified three days after TK release provide further evidence that this TK application (400 mmHg for 5 h) was sufficient to induce profound muscle injury. Interestingly, we also found cell degeneration and necrosis in the bone marrow. Relatively little attention has been directed towards the impact of TK-induced ischemia on bone. Kennedy et al. [22] reported a complete cessation of blood flow in the bone and bone marrow following TK application in dogs. This would explain the extensive damage to the bone marrow following TK in the present study. While there have been reports of osteonecrosis in response to ischemia [23,24], to our knowledge, this is the first report of the resultant histological damage to bone marrow following prolonged (5 h) TK application.

AKI develops in 10% to 40% of patients with severe rhabdomyolysis [25], and one of the well-established mechanisms producing renal injury is reduced perfusion (pre-renal injury). For example, intravascular volume loss due to fluid sequestration within damaged muscle fibers or the interstitium decreases MAP and renal perfusion [26], which stimulates sympathetic nervous and renin–angiotensin system activities. These changes can together cause an acute decrease in GFR. In the current study, acute pre-renal injury was found at 1.5 h after TK release, as the decrease in GFR was mild, transient, and recovered when MAP was normalized. Notably, the change in MAP was concomitant with an approximate 10% increase in Hct, suggesting that the hypotension was due at least in part to water loss from the vasculature to surrounding tissues. In addition, the 10% increase in Hct may directly reduce blood pressure via increasing shear stress-induced vasodilation [27], and thus further reduce renal perfusion. Meanwhile, damaged skeletal muscle may release multiple vasoconstrictors such as myoglobin, endothelin-1, thromboxane A2, and reactive oxygen species [28,29,30], which can simultaneously reduce renal perfusion and GFR. For example, direct infusion of myoglobin was shown to decrease blood flow and oxygen partial pressure in the outer medulla in healthy rats [31]. 

Interestingly, in the time control group, the GFR was transiently increased during the recovery period (1.5 h after stopping general anesthesia) when compared to its BL. A possible explanation is that a “reactive hyperemia” might have occurred when the animals were recovering from the suppression of cardiopulmonary function and renal perfusion induced by the 5 h of anesthesia [32,33]. If this is the case, the decrease in GFR in the TK group may be underestimated. However, the average creatinine clearance rates within the first 24 h were not different between the groups, suggesting that these transient changes in GFR observed at 1.5 h in both groups were both well compensated within the 24 h window. The average creatinine clearance rate from 24 to 48 h was slightly lower in the TK group compared to that in the CON group, but the levels were not different from their baselines and were still within the normal range [34].

In addition to hemodynamic impacts on the kidney, myoglobin may directly damage the renal tubules due to its toxic effect. Myoglobin is rapidly filtered through the glomeruli and reabsorbed in the proximal tubules, where iron is taken up by ferritin, which is less toxic [11]. During rhabdomyolysis, filtered myoglobin overwhelms the kidney’s ability to detoxify the iron, leading to the accumulation of ferrihemes and free radicals and resulting in acute tubular injury [35]. While we did not measure myoglobin levels directly in this study, increases in plasma CK levels are an accepted marker of myoglobin release from injured muscle [11]. However, despite the massively increased CK levels produced by prolonged TK application, there was no histological evidence for intrarenal injury, and the GFR was only transiently decreased. It should be noted that the rats increased their water consumption post-TK application, presumably due to dehydration, as indicated by increased Hct. Increased urinary flow and decreased tubular uptake may accelerate myoglobin excretion and thus minimize its impact on the kidney. Indeed, TK rats exhibited a three-fold increase in urinary output, concomitant with a marked increase in urine protein excretion within 24 h after TK. Such urination occurred spontaneously without parenteral volume resuscitation and did not cause further dehydration, as MAP and Hct were both normalized within 24 h. Similarly, a recent study showed that 4 days after glycerol injection, rats exhibited rhabdomyolysis and increased fractional excretion of sodium and urinary volume [36]. Notably, in patients with rhabdomyolysis syndrome, urine output usually decreases, which further exacerbates the accumulation of myoglobin and formation of casts in the renal tubules [37]. For this reason, increasing urine output with fluid resuscitation or diuretics is a critical therapy to alleviate renal injury in humans [37]. 

Moreover, increasing urine pH, such as by using sodium bicarbonate as a buffer, is another important strategy to prevent rhabdomyolysis-induced AKI [38]. This is because the toxicity of myoglobin to renal tubules is facilitated by an acidic environment in which the globin chain can dissociate from the iron-containing ferrihemate portion [39]. Interestingly, we found that the urine pH was higher from 24 to 48 h after TK release without any treatment with bicarbonate salts. Given that the kidney has a strong pH buffering capacity with over 85% of filtered HCO_3_^−^ being reabsorbed, the spontaneous urine alkalinization was likely due to suppressed reabsorption of HCO_3_^−^, which was echoed by the significant decrease in circulating HCO_3_^−^ on the third day after TK release. However, more studies are needed to confirm this protective mechanism. 

AKI is defined “by an abrupt decrease in kidney function that includes, but is not limited to, acute renal failure” [40]. The clinical definition of AKI is based on serum creatinine levels (≥1.5 times the baseline) or decreased urine output for 6 h [40]. Blood creatinine levels in the current study were 185% higher than BL values at 1.5 h following TK release but decreased to BL levels by 48 hrs. By the clinical definition, AKI existed at 1.5 h following TK release. However, creatinine is measured and used in clinical patients due to the inability to measure GFR directly, and elevated creatinine is recognized to not always represent true reductions in GFR [41]. In fact, the daily creatinine clearance rate was unchanged with TK treatment, providing direct evidence that the elevated plasma creatinine was more likely due to increased creatinine generation rather than impaired renal clearance. It should be realized that, in addition to the fact that creatinine released from injured muscle could profoundly contribute to elevated creatinine levels, the clearance of creatinine is very slow (from several hours to days) compared to the sinistrin used for GFR measurement (within 30 min) [42,43]. Therefore, creatinine levels could still be higher than normal even though renal function is normalized. In this laboratory study, direct measurement showed a decrease in GFR 1.5 h after TK release, but this was normalized by 24 h, a time at which creatinine was still elevated. Taken together, this leads to the conclusion that there was some decrease in renal function immediately following TK release but that this was mild and reversible. In sum, the rodent model was able to reproduce clinical signs observed in rhabdomyolysis in human patients, such as significant increases in creatinine, volume depletion (third spacing), hemoconcentration, hypotension, and impairment of renal function. Additionally, by increasing water consumption in response to volume loss in the circulation, the rats were able to volume-load and compensate for these initial impairments, just as volume resuscitation provided to patients improves renal function following rhabdomyolysis [3]. This study is not without its limitations. First, we only measured CK, MAP, and GFR at 1.5 h after TK release in order to allow full recovery from anesthesia. The actual time points for the peak changes in these parameters are therefore unclear. However, this limitation does not affect our conclusion since the average 24 h creatinine clearance was similar in both groups. Another limitation is the lack of a hemorrhage component. In terms of operational relevance for TK application, it would be expected that significant blood loss might occur before effective TK application. This is even truer in instances where bilateral TKs would be required. It is possible that the addition of hemorrhage may provide a “double hit” that would be more likely to result in AKI due to decreased renal perfusion pressure associated with hypotension. However, it should be noted that TK is often applied on the battlefield too liberally, even without significant hemorrhage, as is currently occurring in Ukraine [4].

In summary, we established a rat model of rhabdomyolysis induced by prolonged TK treatment. However, the massive rhabdomyolysis only resulted in an early, transient decrease in GFR and was not associated with pathological damage to the kidney assessed 3 days later. This lack of acute intrarenal damage differs from less physiologically relevant (e.g., glycerol injection) animal models of rhabdomyolysis, in which both functional and anatomical kidney damage is a frequent consequence. To our knowledge, the current study is the first to show that rats can increase urinary flow and pH during rhabdomyolysis, which may be responsible for the minimized renal injury. In addition, our finding of bone marrow necrosis also suggests that more attention should be paid to the effects of prolonged TK application on bone health, a relatively understudied phenomenon.

## Figures and Tables

**Table 1:** Selected blood and plasma measures in control (CON) and tourniquet (TK) groups across time.

	Group	Baseline	1.5 h	24 h	48 h	72 h
Sodium (mmol/L)	CON	141	140	141	140	140
		(2)	(3)	(2)	(1)	(1)
	TK	138 *	135 *^#^	136 *	140	137 *
		(2)	(1)	(2)	(1)	(3)
Calcium	CON	1.37	1.28	1.37	1.29	1.34
(mmol/L)		(0.07)	(0.24)	(0.06)	(0.10)	(0.19)
	TK	1.33	1.26	1.22	1.31	1.12 *
		(0.19)	(0.03)	(0.08)	(0.10)	(0.27)
pH	CON	7.45	7.45	7.44	7.49	7.39
		(0.03)	(0.06)	(0.02)	(0.02)	(0.08)
	TK	7.44	7.40 *	7.48	7.42 *	7.39
		(0.02)	(0.05)	(0.08)	(0.04)	(0.08)
Lactate	CON	1.09	1.10	1.04	0.84	0.93
(mmol/L)		(0.54)	(0.63)	(0.63)	(0.39)	(0.33)
	TK	1.02	2.00 *^#^	1.86 *^#^	1.49 ^#^	1.83 *^#^
		(0.36)	(0.37)	(0.63)	(0.63)	(0.48)
pCO_2_	CON	43.6	45.7	44.2	36.2	48.2
(mmHg)		(5.2)	(6.1)	(2.7)	(4.5)	(9.8)
	TK	43.8	46.5	37.5	44.3	39.3 *
		(4.5)	(9.4)	(12.2)	(4.9)	(10.6)
BUN (mg/dL)	CON	14.6	12.9	14.8	13.9	12.3
		(3.8)	(3.2)	(1.8)	(3.6)	(12.3)
	TK	16.6	31.0 *^#^	24.7 *^#^	15.4	14.8
		(2.0)	(5.1)	(8.1)	(2.1)	(3.6)
Cr (mg/dL)	CON	0.257	0.329	0.320	0.229	0.314
		(0.053)	(0.111)	(0.084)	(0.095)	(0.038)
	TK	0.300	0.857 *^#^	0.517 *^#^	0.260	0.267
		(0.076)	(0.162)	(0.331)	(0.055)	(0.103)
BUN/Cr	CON	61.4	43.8	50.3	76.9	38.8
		(15.0)	(17.5)	(20.9)	(57.0)	(10.5)
	TK	58.9	37.3	55.9	61.7	58.3*
		(16.8)	(9.8)	(16.7)	(17.3)	(11.6)

Time points represent baseline, 1.5 h post-bilateral TK release, and 24 h, 48 h, and 72 h post-TK application. Data represent mean (SD). Statistical analysis was performed using a two-way repeated ANOVA with a Tukey’s multiple comparison test. * Significantly different (*p* < 0.05) from CON at the same time point. ^#^ Significantly different (*p* < 0.05) from baseline value within the same group. The respective n for each group at BL, 1.5 h, 24 h, 46 h, and 72 h was 7, 7, 5, 7, 7 for CON and 8, 7, 6, 5, 6 for TK.

## Data Availability

The raw data supporting the conclusions of this article will be made available by the authors on request.

## Supplementary Materials

The following supporting information can be downloaded at https://www.mdpi.com/article/10.3390/biomedicines12112607/s1.
