# Supplementary figures and images for: Rhabdomyolysis After Prolonged Tourniquet Application Is Associated with Reversible Acute Kidney Injury (AKI) in Rats
Source: Biomedicines. 2024 Nov 14;12(11):2607. doi: 10.3390/biomedicines12112607 (PMC11592118; doi:10.3390/biomedicines12112607)

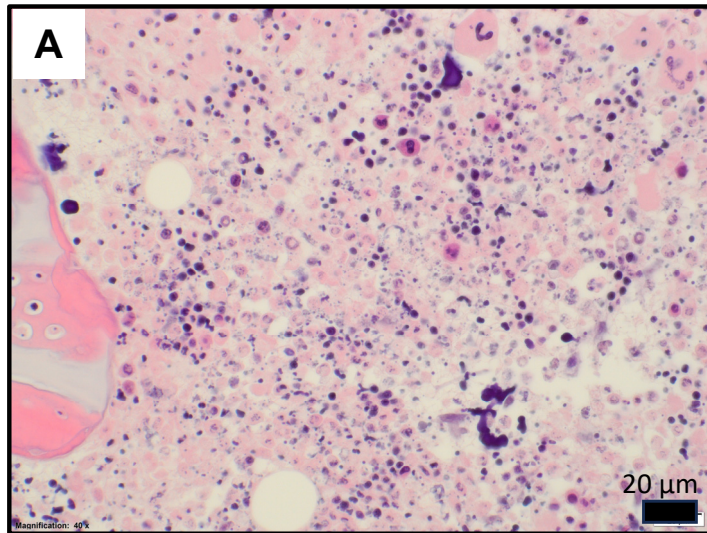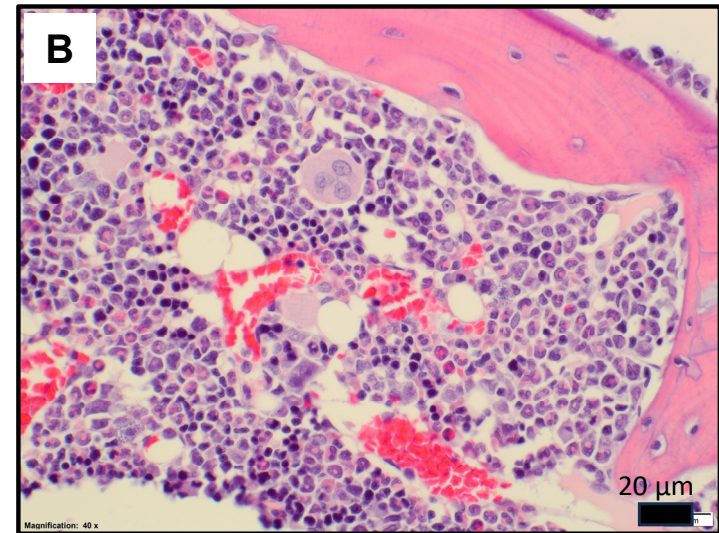

Supplemental Figure 1

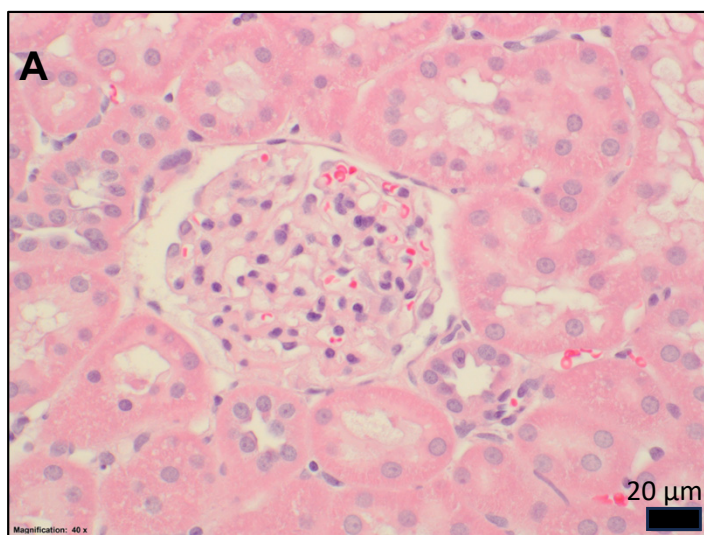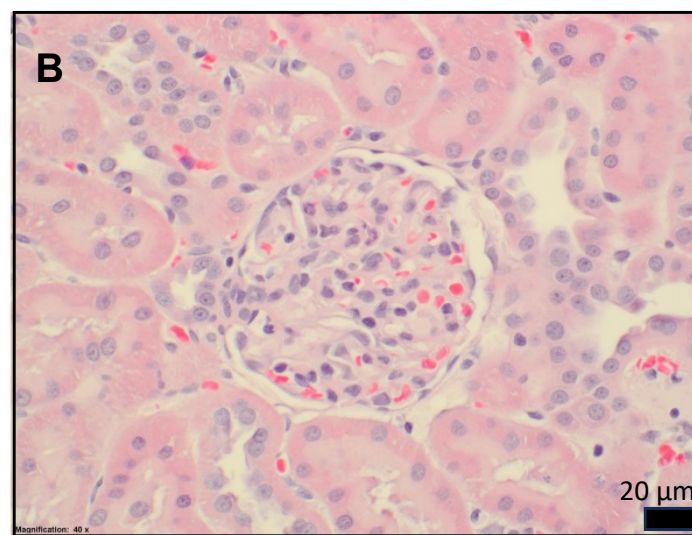

Supplemental Figure 2

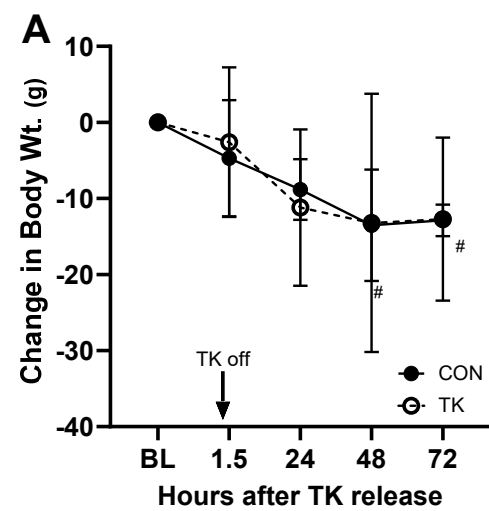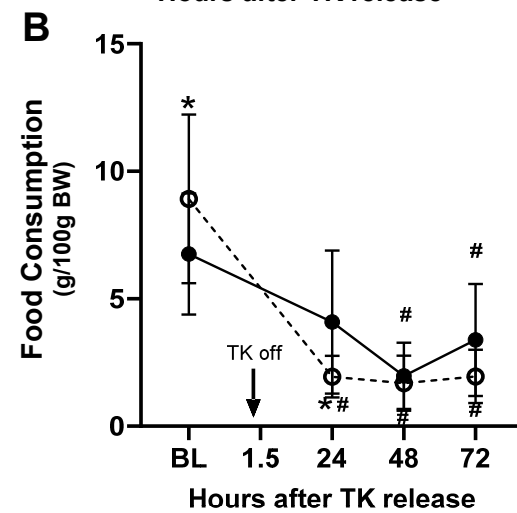

Supplemental Figure 3

Supplement: Supplementary file 1 [file biomedicines-12-02607-s001.zip › biomedicines-3245174-supplementary.pdf]
